# Catching a wave

**DOI:** 10.7554/eLife.21236

**Published:** 2016-10-11

**Authors:** P Michelle Fogerson, John R Huguenard

**Affiliations:** 1 Department of Neurology and Neurological Sciences, Stanford University School of Medicine, Stanford, United Statesfogerson@stanford.edu; 1 Department of Neurology and Neurological Sciences, Stanford University School of Medicine, Stanford, United StatesJohn.Huguenard@stanford.edu

**Keywords:** oscillations, activity depedent development, retinal waves, plasticity, EEG, Rat

## Abstract

Temporary circuits amplify spontaneous activity in the visual system of neonatal rats.

**Related research article** Murata Y, Colonnese MT. 2016. An excitatory cortical feedback loop gates retinal wave transmission in rodent thalamus. *eLife* **5**:e18816. doi: 10.7554/eLife.18816

As the brain develops, neurons start to form the sensory networks that enable us to interpret the world around us in terms of light, sound and touch (Huberman et al., 2008). Before these sensory networks are fully functional, sensory structures such as the retina and cochlea create waves of spontaneous neural activity that help to shape the network (Kirkby et al., 2013). At the same time, the visual cortex produces spontaneous oscillations called spindle-bursts that depend on input from the retina (Hanganu et al., 2006). These various forms of spontaneous activity only occur during a short developmental window. In rats, for instance, they are only produced in the retina and visual cortex within the first two weeks after birth, just before the young rat can open its eyes at postnatal day 14.

In the visual system, a temporary circuit in the retina produces spontaneous retinal waves that sweep across its surface, and output neurons transmit these waves through the optic nerve to the visual thalamus (Galli and Maffei, 1988). Thalamic axons then project this information to the visual cortex, completing the basic visual pathway. It has taken over twenty years of research to figure out the details of the temporary circuit that produces retinal waves (Ford and Feller, 2012), but we know relatively little about the circuits that transform retinal waves, which are poorly synchronized, into spindle-bursts, which are highly synchronized, in the thalamus and cortex. Now, in *eLife*, Yasunobu Murata and Matthew Colonnese of George Washington University have shed new light on this problem (Murata and Colonnese, 2016).

In experiments performed on neonatal rats, Murata and Colonnese mapped the roles of the retina, thalamus and cortex in the creation of spindle-bursts by removing these three regions in the brain's visual pathway one by one and recording any neural activity that remained elsewhere in the pathway. Silencing the retina (by injecting it with activity-blocking drugs) reduced activity in both thalamus and cortex by 90%, showing that retinal waves drive spindle-bursts throughout the visual pathway. By contrast, silencing the thalamus reduced the level of spontaneous activity in the cortex and completely prevented the synchronization of any remaining activity there. It is clear, therefore, that the thalamus is required to convert retinal waves into spindle-bursts in the cortex. Finally, silencing the cortex nearly abolished spontaneous activity in the thalamus, leaving only weak and slow residual oscillations.

Taken together, these results suggest that a pathway that connects the cortex and thalamus both amplifies and synchronizes oscillations generated in the thalamus to produce spindle-bursts in response to retinal waves. However, this synchronizing circuit is active only for a short time: this period, which is called the spindle-burst window, lasts from postnatal day 5 to postnatal day 11.

What circuit changes underlie this transient amplifier? To tackle this question, Murata and Colonnese used optogenetic techniques to stimulate the cortex and mimic spontaneous spindle-bursts in the corticothalamic pathway. They found that, early in the spindle-burst window (postnatal day 5–7), the pathway amplified thalamic activity by a small amount, but did not synchronize it. Later, when the pathway had been strengthened (postnatal day 9–11), repeated stimulation of the cortex led to spindle-burst-like oscillations in the thalamus. Then, after the spindle-burst window had closed (postnatal day 13–14), cortical stimulation excited and then suppressed thalamic output, preventing spindle-burst-like oscillations in thalamus. These findings suggest that, later in development, the cortex inhibits the thalamus and prevents the corticothalamic pathway from amplifying spindle-bursts.

So how does the cortex inhibit the thalamus and shut down the transient amplifier? In adults, the corticothalamic pathway directly excites the sensory thalamus and, at the same time, indirectly inhibits it by activating a feedforward pathway through the thalamic reticular nucleus (Figure 1). Because indirect inhibition outweighs direct excitation, activation of the corticothalamic pathway can truncate the output from the thalamus (Golshani et al., 2001). The late development of this feedforward pathway might explain when the transient amplifier is shut down.

**Figure 1.**
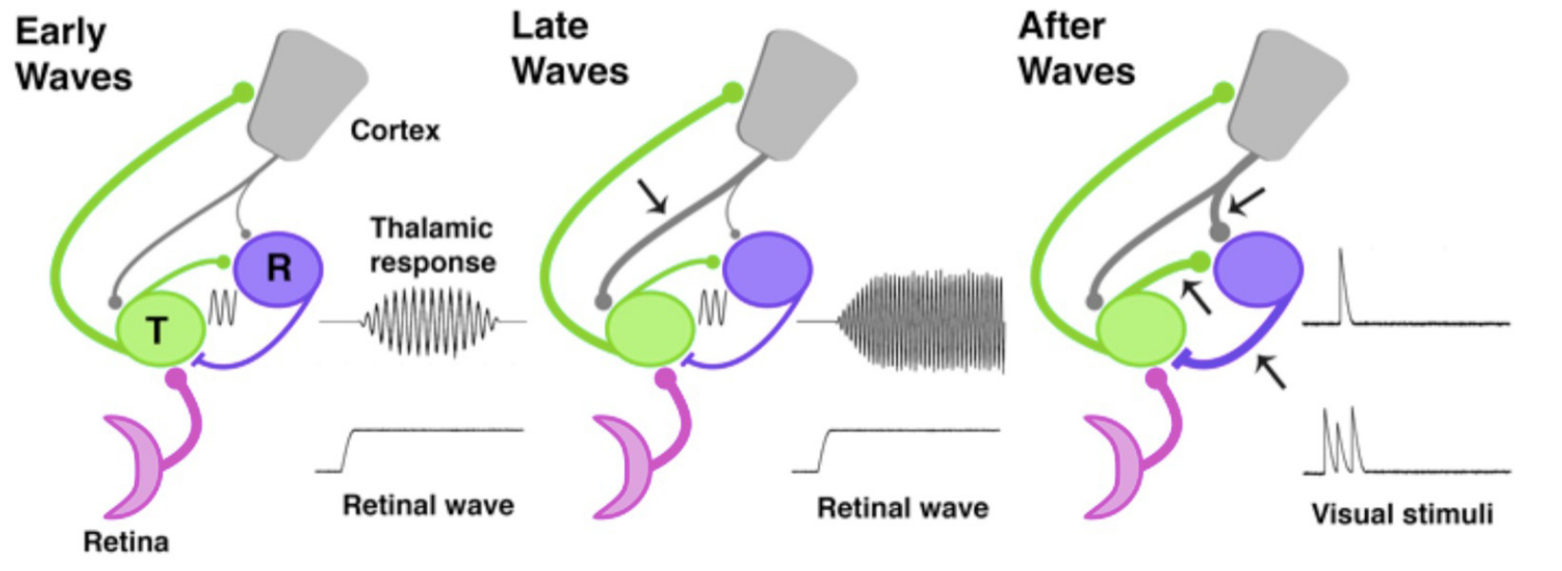
The development of thalamic circuits. Here, we show how developing thalamic and cortical pathways might temporarily enable spindle-bursts. Pathways linking the retina (pink), thalamus (green, T), reticular thalamic nucleus (purple, R), and cortex (grey) are shown at three different times during development based on data from rats and mice. The thickness of each line represents the strength of the pathway; arrows mark pathways that increase in strength at each developmental timepoint. Transmission speed and the strength of the connection between the thalamus and its synaptic partners help to set the frequency and level of synchronization of oscillations (Bal et al., 1995). During the early and middle stages of the spindle-burst window (left), an ascending pathway transmits retinal waves to thalamus and cortex. Early in the spindle-burst window, thalamic circuitry produces brief, low frequency spindle-bursts (indicated by sine wave), likely through connections to the reticular nucleus, which have just become functional at this time. A modest corticothalamic projection (grey) amplifies this oscillation. Later in the spindle-burst window (middle), a strengthened corticothalamic projection (arrow) amplifies, speeds, and prolongs thalamic spindle-bursts. After the spindle-burst window (right), reciprocal connections between thalamus and the reticular nucleus continue to develop (lower arrows), as may the projection from the cortex to the reticular nucleus (upper arrow). At the same time, both the thalamic and cortical components of spindle-bursts disappear: this enables the thalamus to respond to transient stimulation from the retina, before the thalamic response is quickly suppressed by the cortex.

In addition to spontaneously generated retinal waves, visual stimuli that excite the retina can also evoke a spindle-burst in the thalamus and cortex, but only late during this brief developmental window (postnatal day 9–11). Using light to stimulate the retina, Murata and Colonnese find that visually-evoked spindle-bursts are similarly amplified by the corticothalamic pathway. After the spindle-burst window closes, visually-evoked responses in the thalamus are no longer synchronized, even after the cortex has been inactivated: this shows that the thalamic component of spindle-bursts has also disappeared by this time. Shutting down thalamic spindle-burst responses to visual input is essential for visual processing in adults.

During the spindle-burst window, thalamic neurons are rapidly developing, which may temporarily enable spindle-bursts. Their electrical excitability increases, as does synaptic connectivity with the reticular nucleus (Figure 1), both of which contribute to mature thalamic circuitry (Warren and Jones, 1997). The thalamus can only produce spindle-bursts for a few days during development, and during this time it is only partially wired into oscillatory networks with the reticular nucleus and cortex.

Synchronized neural activity during development can influence the shape of mature neural circuits. During the second postnatal week in mice, synapses in the cortex that fire out of sync with their neighbors become weaker, while synapses that fire in sync persist (Winnubst et al., 2015). Synchronized spindle-bursts, both spontaneous and sensory-evoked, may have a similar effect on synaptic connections because they provide a way to coordinate neuronal firing patterns, which is required to stabilize synapses.

Experiments that distort retinal waves by targeting their circuit generator have shown how they shape connectivity patterns between the retina and thalamus (Kirkby et al., 2013). Now that we have a better understanding of the circuits that connect the retina, the visual thalamus and the visual cortex, experiments that disrupt spindle-bursts could lead to further insights into the development of the visual system.
